# Bridging the Healthcare Gap: A Mixed-Method Study on the Impact of Ayushman Bharat Health Account (ABHA) on Underprivileged Populations of Bhopal and Raisen Districts

**DOI:** 10.7759/cureus.92272

**Published:** 2025-09-14

**Authors:** Manju Toppo, Devendra Gour, S K Patne, Neeta Kumar, Rashmi Pilkhwal, Siddharth Kimtee, Kiranmai Pandi, Gursharan Singh Mehta, Nilufar Mondal, Khushboo Gupta

**Affiliations:** 1 Community Medicine, Gandhi Medical College, Bhopal, IND; 2 ICMR Headquarters, Indian Council of Medical Research (ICMR), New Delhi, IND; 3 Community Medicine, Hamidia Hospital, Bhopal, IND

**Keywords:** abha, ayushman bharat digital mission, ayushman bharat health account, healthcare delivery system, underprivileged, urban and rural community

## Abstract

Background and objectives: The Ayushman Bharat Health Account (ABHA) represents a significant advancement in India's healthcare infrastructure, particularly aimed at enhancing access to medical services for underprivileged populations in both rural and urban settings. The study aimed to evaluate the awareness and understanding of the ABHA among underprivileged populations in both rural and urban settings, to analyze the attitude of the community toward digital health records and the ABHA system, and to analyze the relationship between the status of ABHA and various sociodemographic characteristics of the study population.

Materials and methods: A mixed-method study was conducted in Bhopal as part of the baseline survey for the multi-centric study under the Indian Council of Medical Research (ICMR) Task Force Project titled "Task force study for evaluation of community level acceptability, scalability and linkage within the health system of ICMR pre-validated Labike technologies for screening & diagnosis in rural and urban population - An Implementation research". A door-to-door household survey was conducted among a selected underprivileged population living in both rural and urban areas. Categorical variables were expressed as frequencies and percentages, and chi-square was calculated to study the association between sociodemographic characteristics, such as gender, age, residence, education, and employment, and ABHA status, and thematic analysis was conducted for qualitative data.

Results: The study's results illustrate the distribution of participants by various sociodemographic characteristics among 5709 participants. A total of 2460 (43%) of the participants were females, and the majority of the participants (2187, 38.3%) belonged to the 15-30 years of age. In this study, only 96 (3.4%) and 66 (2.3%) of residents of rural and urban areas of central India had ABHA, and this difference was statistically significant. Unlinked Aadhaar numbers (40.6% rural vs. 51.2% urban), a shortage of mobile phones, and poor awareness levels, which revealed notable differences between rural and urban populations, were the main obstacles to the generation of ABHA. This study assessed the relationship between a number of sociodemographic factors and the generation of an ABHA. The factors that significantly influence the likelihood of ABHA generation are gender (χ² = 11.6, p < 0.05), place of residence (χ² = 5.8, p = 0.01), educational status (χ² = 4.4, p = 0.03), and employment status (χ² = 14.4, p < 0.0001), as evidenced by the statistically significant association found between these factors and ABHA generation.

Conclusions: The study found many obstacles despite the promising framework that ABHA developed while evaluating awareness and understanding of the ABHA among underprivileged populations in both rural and urban settings. The study analyzed community attitudes toward the ABHA system through in-depth interviews, which found four major themes: (1) unavailability of mobile phones; (2) Aadhaar not linked to mobile numbers; (3) not willing to get ABHA number; (4) unaware of ABHA and no interest in getting it. This study also assessed the relationship between the status of ABHA and various sociodemographic characteristics of the study population, in which gender, residence, education, and employment were found to be statistically significant.

## Introduction

The Ayushman Bharat Health Account (ABHA) represents a significant advancement in India's healthcare infrastructure, particularly aimed at enhancing access to medical services for underprivileged populations in both rural and urban settings. Launched as part of the Ayushman Bharat Digital Mission (ABDM), ABHA is designed to create a comprehensive digital health ecosystem that facilitates the efficient exchange of health information and improves the quality of care for millions of citizens [1].

The introduction of the ABHA card complements these efforts by enabling beneficiaries to maintain a digital record of their health information, thereby streamlining access to healthcare services across various providers [2]. It serves as a unique 14-digit identifier that links an individual's health records within the ABDM framework. Authorized parties can access vital health records without requiring physical documentation by just sharing their ABHA identity (ID) with healthcare professionals [3,4]. This guarantees continuity of care and minimizes treatment delays, particularly when patients change providers or seek a second opinion [5-7].

By connecting insurance policies and health accounts to the ABHA ID, hospitals may easily get policy information for cashless claims processing [8-10]. Thus, ABDM is envisioned as a compelling, patient-centered supplement to conventional healthcare delivery methods [11-15].

This mixed-method study focuses on evaluating the awareness, acceptance, and utilization of the ABHA among selected underprivileged communities in both rural and urban settings, analyzes the attitude of the community toward digital health records and the ABHA system, and examines the relationship between the status of ABHA and various sociodemographic characteristics of the study population. The findings will contribute valuable data to inform policymakers and stakeholders about the effectiveness of digital health initiatives in bridging gaps in healthcare delivery, ultimately aiming for improved health outcomes and enhanced quality of life for underprivileged populations in India.

## Materials and methods

**Figure 1:**
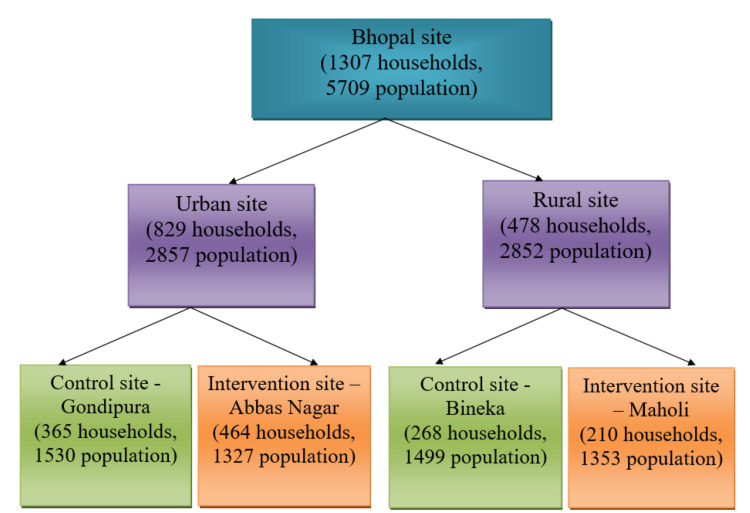
Number of households and participants covered in both rural and urban sites.

As part of the baseline survey for the multi-centric study under the Indian Council of Medical Research (ICMR) Task Force project titled "Task Force study for evaluation of community level acceptability, scalability and linkage within the health system of ICMR pre-validated Labike technologies for screening & diagnosis in rural and urban population - An Implementation research," a door-to-door household survey was conducted among a selected underprivileged population, i.e., socially and economically disadvantaged groups residing in rural areas (Bineka and Maholi) and urban areas (Gondipura and Abbas Nagar), as depicted in Figure 1, who face limited access to quality healthcare services. As the main Task Force study is an implementation research with a cluster randomized controlled design in site allocation, one intervention and one control arm have been randomly selected in both rural and urban areas.

Study design

A mixed-method study was done in both rural and urban areas of the Bhopal site.

Study setting

With a predefined total sample size of 35,000 (5,000/site), the study was undertaken in six study locations: Varanasi, Muzaffarpur, Guwahati, Chennai, Srinagar, and Bhopal.

Sample size

Quantitative

The study participants were required to be chosen from the designated impoverished rural and urban areas of 5000 populations (i.e., 1000 households assuming an average household size of five individuals), including 2500 for intervention and 2500 for control sites to cover the sample size (Figure 1).

The sample size of 2500 per area was calculated in the main Task Force study "Task Force study for evaluation of community level acceptability, scalability and linkage within the health system of ICMR pre-validated Labike technologies for screening & diagnosis in rural and urban population - An Implementation research," using the design of implementation studies for quality improvement programs and derived formulas for sample size in implementation research by Cheung and Duan [16].

Kaur et al. [17] recently published a cost reduction strategy for comprehensive healthcare in the community. If the share of treatment for patients with newly diagnosed, uncomplicated diabetes or hypertension at health and wellness centers increases to 70%, from the existing 4% at subcenters and primary health centers, the annual population-based screening becomes a cost-saving strategy. This Task Force study aims to document the baseline situation and end-term utilization level after one year of follow-up (Table 1).

**Table 1:** Sample size calculation.

Preselected values				
α	One-tailed	Two-tailed	1-β	Zβ
0.1	1.282	1.645	0.7	0.524
0.05	1.645	1.96	0.8	0.842
0.025	1.96	2.241	0.9	1.282
0.01	2.326	2.576	0.95	1.645
0.001	3.09	3.291	0.975	1.96

The sample size was determined based on a one-tailed test, with a significance level (α) of 0.01 and power (1-β) of 95%, resulting in a required sample size of approximately 2,326 participants per group. To account for potential dropouts and non-response, this was rounded to 2,500 participants per group (Table 1).

Qualitative

In-depth interviews were conducted using a pre-designed, pre-tested, and pre-validated questionnaire. Data collection was done until saturation was reached. A total of 20 responses were collected.

Study duration

The study was conducted for a period of six months from November 2022 to May 2023.

Study participants

The target population consisted of underprivileged individuals and families eligible for the Ayushman Bharat scheme. The permanent residents of the area who provided informed consent to participate in the study were recruited. The responses were sought from the heads of the households only; for households where the head was not available, responses were sought from available adult family members.

Study tools

The survey was conducted using a pre-designed, pre-validated questionnaire. The questionnaire included sociodemographic details of the participants, the presence of ABHA numbers, and reasons for not having an ABHA ID. In-depth interviews were also done to analyze the attitude of the community toward ABHA.

Statistical analysis

Data were entered into a Microsoft Excel sheet. The statistical analysis was performed using Epi Info 7 (Centers for Disease Control and Prevention, Atlanta, GA). To examine the potential associations between sociodemographic characteristics and ABHA status, a chi‑square test was conducted. Variables with a p-value of less than 0.05 were considered statistically significant. Thematic analysis was done for qualitative data.

Ethical clearance

Ethical clearance was obtained from the Institutional Ethics Committee, Gandhi Medical College, Bhopal (Letter No. 6699/MC/IEC/2022; Dated: 15/02/2022).

## Results

**Figure 2:**
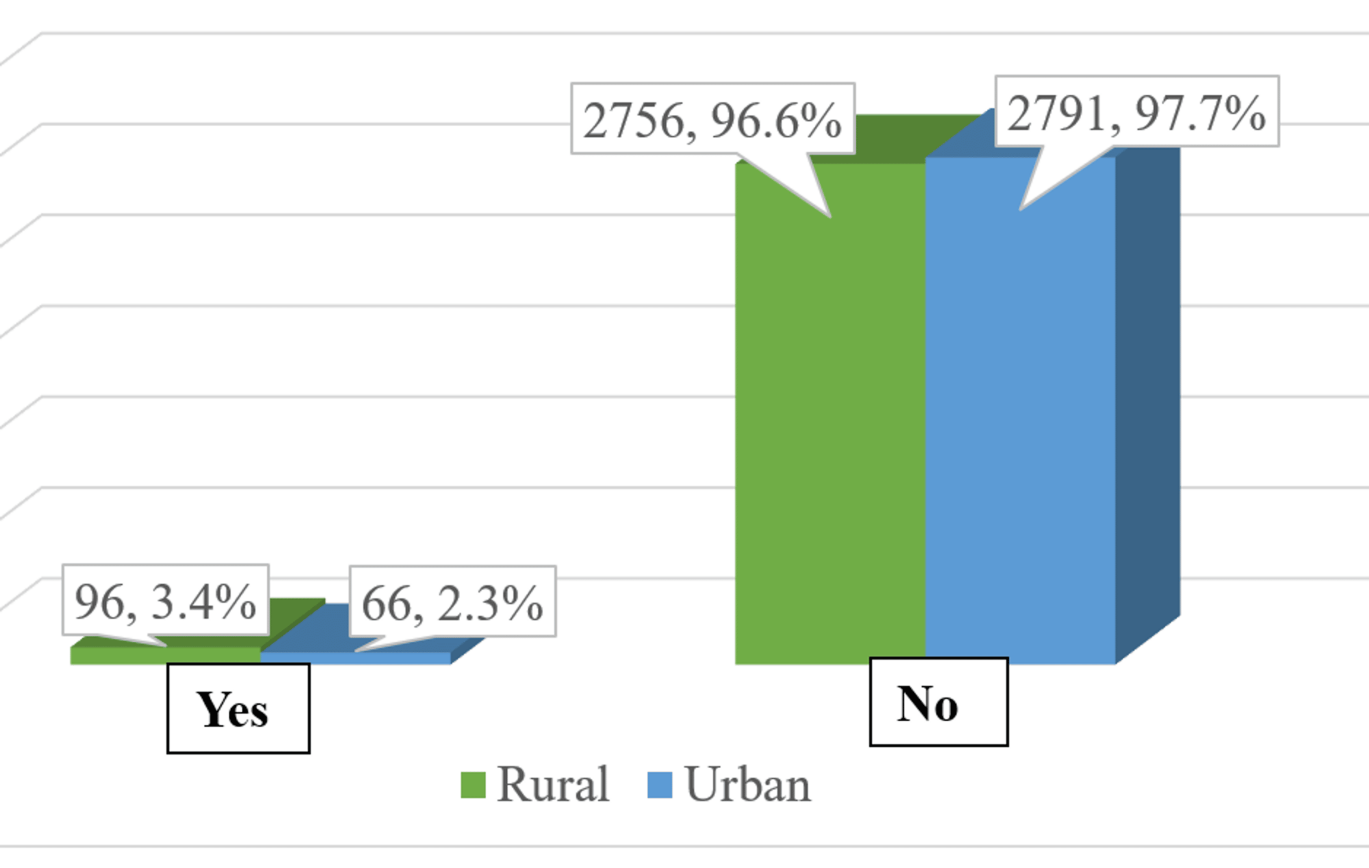
Distribution of participants with and without Ayushman Bharat Health Account (ABHA).

The study's results illustrate the distribution of participants by various sociodemographic characteristics among 5709 participants. A total of 2460 (43%) of the participants were females, and the majority of the participants (2187, 38.3%) were 15-30 years of age. A total of 2857 (50.1%) of the participants resided in urban areas compared to 2852 (49.9%) living in rural areas. The family structure was predominantly nuclear, with 714 households (54.6%), while joint families accounted for 593 households (45.4%). In terms of education, there were more literate participants, with a total of 3678 individuals (64.4%) compared to 2031 illiterate individuals (35.6%). Regarding employment status, a majority of the respondents were employed or engaged in skilled/semi-skilled/unskilled work, totaling 3988 individuals (69.9%). Conversely, those categorized as unemployed, homemakers, or students numbered 1721 individuals (30.1%). Only 96 (3.4%) and 66 (2.3%) participants from rural and urban areas, respectively, had ABHA (Figure 2).

The reasons for not having an ABHA among respondents from rural and urban areas highlight the disparities in access and awareness across these populations. In rural areas, 457 (16.6%) of respondents reported that a mobile phone was not available, while 419 (15%) of urban respondents cited the same issue. A significant barrier for both populations was the availability of Aadhaar, which is essential for obtaining an ABHA number. In rural areas, 226 (8.2%) of individuals lacked Aadhaar, compared to 204 (7.3%) in urban areas. A larger proportion of respondents indicated that while they had Aadhaar, it was not linked to their mobile number, with 1119 (40.6%) in rural areas versus 1429 (51.2%) in urban areas. A notable 582 (21.1%) of rural participants expressed unwillingness to get an ABHA number without any specific reason, compared to just 17 (0.6%) in urban areas. Finally, awareness levels differed significantly: 345 (12.5%) of rural respondents were unaware or not interested in obtaining an ABHA number, while this figure rose to 583 (20.9%) among urban respondents (Table 2).

**Table 2:** Distribution of participants according to the reasons for not having Ayushman Bharat Health Account (ABHA).

Reasons for not having ABHA	Rural, n (%)	Urban, n (%)
Mobile phone not available	457 (16.6)	419 (15)
Mobile phone available, but the internet is not available	6 (0.2)	6 (0.2)
Internet available, yet not able to use; do not know how to use	1 (0.05)	0
Aadhaar is not available, which is a must for the ABHA number	226 (8.2)	204 (7.3)
Aadhaar is available, but is not connected to the mobile number	1119 (40.6)	1429 (51.2)
Do not find any use of getting an ABHA number	14 (0.5)	0
Not willing to get an ABHA number; no specific cause	582 (21.1)	17 (0.6)
Lack of help who can help generate an ABHA number	0	114 (4.1)
Issue of electricity	0	6 (0.2)
May consider getting an ABHA ID in the future if any use is observed	0	0
Not aware of ABHA number; not interested to get it	345 (12.5)	583 (20.9)

The analysis of the factors influencing the generation of ABHA revealed significant differences based on gender, residence, education, and employment status among the respondents. Among females, 91 (3.7%) participants had generated an ABHA, while for males, 71 (2.2%) had generated an ABHA, resulting in a statistically significant difference (χ2 = 11.6, p < 0.05). The age distribution showed that the generation of ABHA varied but was not statistically significant across different age groups. A significant difference was observed based on residence type (χ2 = 5.8, p = 0.01). In rural areas, 96 respondents (3.4%) generated an ABHA compared to 66 (2.3%) in urban areas. The educational background also influenced ABHA generation significantly (χ2 = 4.4, p = 0.03). Among literate respondents, 117 (3.2%) had generated an ABHA as compared to 45 (2.2%) among illiterate respondents. Employment status showed a strong correlation with ABHA generation (χ2 = 14.4, p < 0.0001). Only 27 (1.6%) of those categorized as unemployed, homemakers, or students had generated an ABHA compared to a higher proportion of employed individuals at 135 (3.4%), indicating that employment status significantly affects access to ABHA (Table 3).

**Table 3:** Association between sociodemographic characteristics and Ayushman Bharat Health Account (ABHA) status (N = 5709).

Variable	Category	ABHA generated		Total, n (%)	χ2	df	p
		Yes, n (%)	No, n (%)				
Gender	Female	91 (3.7)	2369 (96.3)	2460 (100)	11.6	1	<0.05
	Male	71 (2.2)	3178 (97.8)	3249 (100)			
Age (years)	0-15	48 (2.2)	2136 (97.8)	2184 (100)	6.1	4	0.15
	15-30	72 (3.3)	2115 (96.7)	2187 (100)			
	31-45	29 (3.5)	805 (96.5)	834 (100)			
	46-60	10 (2.9)	330 (97.1)	340 (100)			
	>60	3 (1.8)	161 (98.2)	164 (100)			
Residence	Rural	96 (3.4)	2756 (96.6)	2852 (100)	5.8	1	0.01
	Urban	66 (2.3)	2791 (97.7)	2857 (100)			
Education	Literate	117 (3.2)	3561 (96.8)	3678 (100)	4.4	1	0.03
	Illiterate	45 (2.2)	1986 (97.8)	2031 (100)			
Employment	Unemployed/homemaker/ student	27 (1.6)	1694 (98.4)	1721 (100)	14.4	1	0.0001
	Employed/skilled/semi-skilled/unskilled	135 (3.4)	3853 (96.6)	3988 (100)			

In-depth interviews were conducted using a pre-designed, pre-tested, and pre-validated questionnaire. Data collection was done until saturation was reached. A total of 20 responses were collected.

The qualitative findings capture community voice and provide insights into their real worries, viewpoints, and obstacles.

Four main themes were identified: (1) unavailability of mobile phones; (2) Aadhaar not connected to mobile number; (3) not willing to get ABHA number; (4) unaware of ABHA and no interest in getting it.

**Figure 3:**
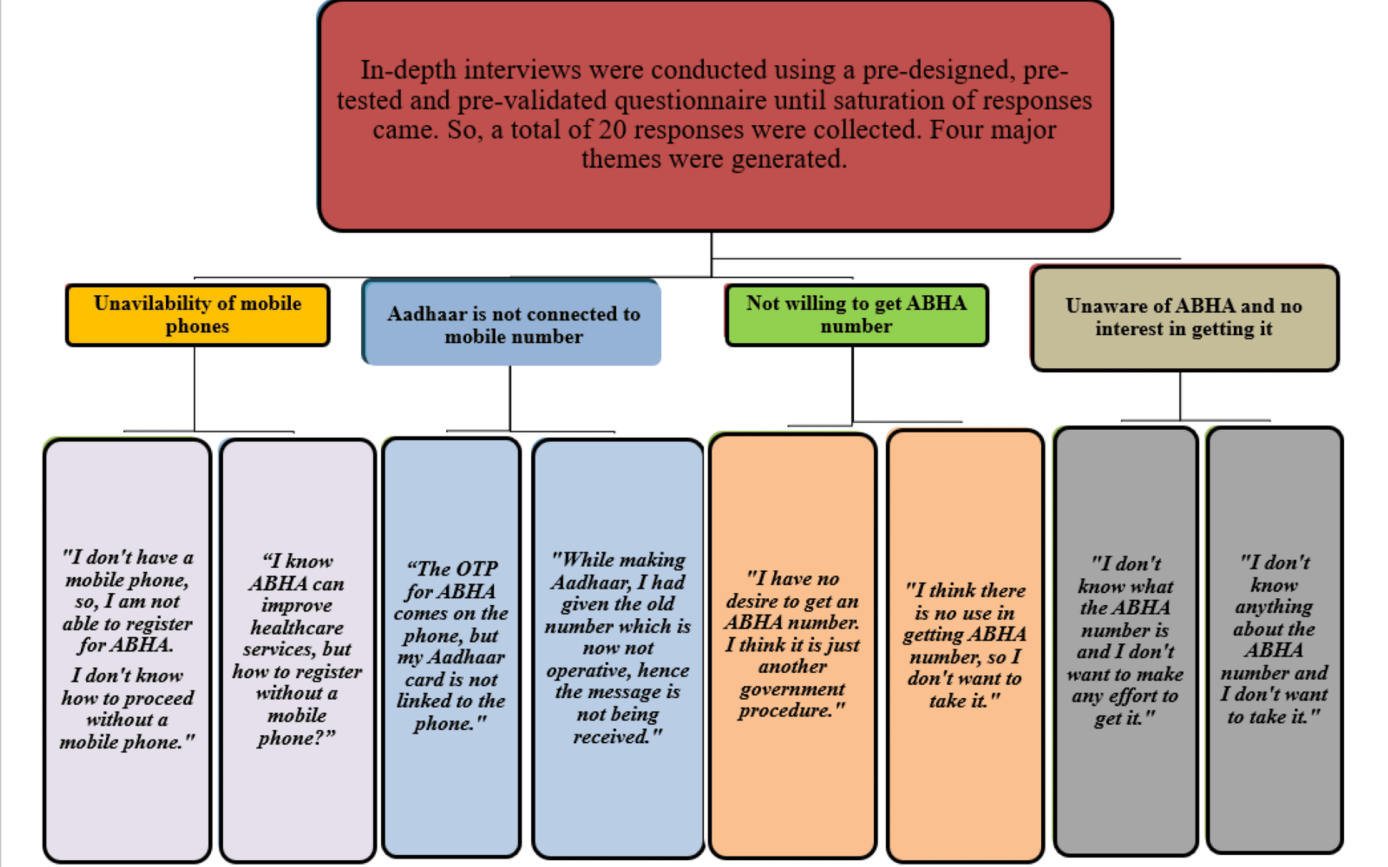
Thematic analysis of community attitude toward ABHA. ABHA: Ayushman Bharat Health Account; OTP: one-time password.

The findings drew attention to structural problems, such as the absence of cell phones and the inability to update Aadhaar with the current number, and behavioral problems, such as ignorance about ABHA, reluctance, and lack of trust (Figure 3).

These findings suggest designated health facilities for ABHA creation so that individuals who do not have mobile phones get a provision to visit local health facilities or centers that would be authorized to assist with the creation and management of ABHA accounts. Staff at these facilities can help them set up the account and access the health information. To generate interest for ABHA, community-level incentives such as health service discounts should be offered to families who sign up for ABHA or use the program's healthcare services.

## Discussion

The Ayushman Bharat initiative, particularly through the ABHA, represents a significant advancement in addressing healthcare disparities in India, especially for underprivileged populations in central India. The digital health ID uptake rate was 78% among the participants from Chandigarh in the study by Gandhi et al. [2] (2024), which is very much higher than in this study. In this study, only 3.4% and 2.3% of residents of rural and urban areas of central India had ABHA, and this difference was statistically significant. In the study by Fazili et al.[11] (2024), a higher proportion of urban respondents (15%) possessed an ABHA, while only 1.4% of rural respondents had an ABHA. In a study by Pangare and Karkar [18] (2024), 62% of participants had ABHA, which is also higher than in the present study. In the current study, 46% of the total participants had Aadhaar, but it was not linked to the mobile number, and this was the major reason for not having an ABHA in this study. This finding is consistent with the findings of Kumar et al.[4] (2023), in which 34.9% of the participants across six sites gave this reason for not having an ABHA. Age-wise distribution of the ABHA shows that the maximum ABHA IDs (1.3%) were generated in the 15-30 years age group, which is in contrast to the findings seen in the study by Pangare and Karkar [18] (2024), where 33.6% of the ABHA holders were in the age group of 31-40 years. The percentage of ABHA among females (3.7%) was found to be higher than among males (2.2%), which was statistically significant too. Still, this gender-based distribution of ABHA deviates from that of the ABHA dashboard [1]. ABHA status of participants was statistically significant and greater among literate respondents (3.2%) than illiterate respondents (2.2%). Compared to the unemployed, homemakers, and students (1.6%), the employed, skilled, semi-killed, unskilled, and professional classes had a somewhat higher percentage of ABHA (3.4%), and it was statistically significant too. ABHA status was better in respondents whose duration of residence in the area was 6-10 years in the study by Fazili et al.* *[11] (2024), whereas in this study, it was better in respondents whose duration of residence in the area was >10 years. In a qualitative study by Kamath et al. [19] (2025), who explored perceptions regarding ABDM among university students, the findings revealed privacy/Aadhaar-linkage concerns and low perceived immediacy of benefit as key barriers, and many preferred to avoid Aadhaar linking. These findings of lack of privacy and perceived utility leading to reduced ABHA creation even among literate/educated subgroups are relevant to the current study, where behavioral barriers (lack of awareness, trust issues, and perceived irrelevance) were present.

Limitations

Both survey responses and qualitative interviews relied on self-reported information. So, recall bias could have been there. While mobile phone and Aadhaar linkage issues were discussed, other crucial digital barriers, such as digital literacy, language barriers, availability of technical help, and access to online services, could not be thoroughly assessed. The study may also overrepresent populations who are non-working/more likely to stay at home, given that surveys were done from people's homes.

Recommendations

The study provides the following recommendations: engaging the community and providing training and skills to improve awareness; informing the community about the benefits and drawbacks of ABHA will help in its better adoption; simplifying communication by creating IEC (information, education, and communication) in the local languages and with cultural relevance; information may be broadly disseminated by utilizing a variety of communication methods, including social media, neighborhood gatherings, and local health camps; outreach initiatives can also be strengthened by working with neighborhood organizations; encouraging government schemes, such as AYUSH, that make it simple for people with mobile phones to manage their medical records; emphasizing elements such as telemedicine consultations, appointment scheduling, and health information access.

## Conclusions

The ABHA is instrumental in addressing healthcare disparities in India by enhancing access, affordability, and quality of care for underprivileged populations. By leveraging digital technology to create a more integrated and patient-centric healthcare system, ABHA aims to ensure that all citizens can receive the necessary health services without financial strain or logistical barriers. As it continues to evolve, ABHA has the potential to significantly improve health outcomes across diverse communities in India. The study focused on the evaluation of the awareness and understanding of the ABHA among underprivileged populations in both rural and urban settings. There are many obstacles to overcome despite the promising framework that ABHA developed. The study analyzed community attitudes toward the ABHA system through in-depth interviews. These interviews found four major themes: unavailability of mobile phones; Aadhaar not linked to mobile numbers; unwilling to get ABHA; and unaware of ABHA and no interest in getting it. Rural regions' limited infrastructure and the disparities in knowledge among various socioeconomic groups prevent the program from reaching its full potential. For ABHA to have the greatest possible impact, focused initiatives to increase community involvement, expedite enrolment procedures, and advance digital literacy are necessary. This study also assessed the relationship between the status of ABHA and various sociodemographic characteristics of the study population, in which gender, residence, education, and employment were found to be statistically significant.
